# A Novel, Possibly Pathogenic, COL4A1 Gene Variant (c.3698G>A) in a Family With Childhood Epilepsy and Leukoencephalopathy

**DOI:** 10.7759/cureus.48553

**Published:** 2023-11-09

**Authors:** Rahul Gaini, Julia Denniss, Ashley Lengel, Elijah A Lackey

**Affiliations:** 1 Neurology, Duke University, Durham, USA

**Keywords:** cerebral imaging, col4a1 gene variant, neurologic disorders, childhood epilepsy, genetic syndromes, variant of unknown significance, collagen disorder, diffuse leukoencephalopathy

## Abstract

This case report explores the clinical presentation and genetic findings of a 44-year-old male with a history of pediatric epilepsy. The patient's daughter, recently diagnosed with autism, underwent genetic testing, revealing a variant of uncertain significance (VUS) in the type IV collagen alpha 1 (COL4A1) gene. The male patient reported a spectrum of neurological symptoms, including chronic migraines, exertional weakness, and sensory disturbances. Detailed neurological examination findings were within normal limits, but a brain MRI unveiled confluent deep white matter T2/fluid-attenuated inversion recovery (FLAIR) signal abnormalities with basal ganglia involvement. Genetic testing identified a novel COL4A1 gene variant, c.3698G>A (p.Gly1233Glu), in the patient, which was also carried by his daughter. The nature and clinical implications of this VUS in the context of the family's clinical history are discussed in this case report, emphasizing the potential significance of this genetic variant in understanding the etiology of the patient's neurologic symptoms. Further research and correlation with clinical findings are needed to elucidate whether this is a pathogenic variant.

## Introduction

This case report describes the case of a 44-year-old male with a unique constellation of symptoms, including pediatric epilepsy, migraines, hemoptysis, and bowel incontinence. A recent diagnosis of autism in his daughter in the setting of abnormal brain imaging and genetic testing prompted him to also undergo brain imaging and genetic testing. Genetic evaluation unveiled a variant of uncertain significance (VUS) in the type IV collagen alpha 1 (COL4A1) gene, a gene implicated in providing instruction for a component of type IV collagen. The VUS was shared by both the patient and his daughter. The discovery of this genetic variant, c.3698G>A (p.Gly1233Glu), raises compelling questions about its potential clinical significance and its role in the family's neurologic symptoms. This report provides a detailed exploration of the patient's clinical presentation, neurological findings, and genetic results, emphasizing the clinical implications of this VUS in the context of the patient's neurologic symptoms. This case also underscores the importance of considering genetic testing in the assessment of complex neurologic conditions and the ongoing quest to unveil the genetic underpinnings of rare neurologic diseases.

## Case presentation

**Figure 1:**
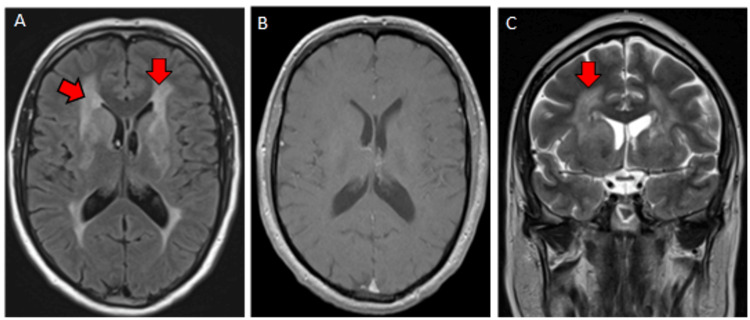
MRI of the brain showing leukoencephalopathy Magnetic resonance imaging (MRI) of the brain shows a pattern of diffuse but asymmetric T2/fluid-attenuated inversion recovery (FLAIR) (panels A and C) deep white matter hyperintensity involving the basal ganglia, including the caudate nuclei, without contrast enhancement on the T1 contrasted sequence (panel B).

A 44-year-old male patient with a history of pediatric epilepsy underwent genetic analysis and brain imaging earlier in the year after his six-year-old daughter, recently diagnosed with autism, was found to have a genetic variant and abnormal brain MRI findings. The patient’s genetic testing uncovered the same variant as seen in his daughter, which was a VUS, in the COL4A1 gene, specifically the presence of c.3698G>A (p.Gly1233Glu). Thereafter, the patient underwent an MRI brain scan, which revealed a confluent increased T2/fluid-attenuated inversion recovery (FLAIR) signal in the supratentorial deep white matter with basal ganglia involvement (Figure 1).

His daughter had similar brain MRI findings. Notably, there were no indications of abnormal enhancement or evolving ischemia. As a result of these findings, the patient was referred to neurology for further evaluation.

On arrival at the neurology clinic, the patient reported a diverse array of neurologic symptoms. Notably, he experienced pediatric epilepsy at the age of one, but his seizures were well-controlled on phenobarbital, which was discontinued later in childhood with no ongoing seizures thereafter. The patient described chronic migraines for years, characterized by an "electrical sensation" to the front of his head and often accompanied by photophobia, phonophobia, and occasional nausea. Additionally, he reported a history of extremity weakness, particularly noticeable in his arms when raising them over his head for an extended period and in his legs after prolonged standing. He also experienced double vision when looking horizontally, particularly to the left. Systemically, the patient also described a chronic cough, occasionally accompanied by hemoptysis, which worsened with eating, drinking, or lying flat. Furthermore, the patient had been struggling with bowel incontinence for several years. The patient had a medical history notable for venous insufficiency in his legs. He was not taking any medications.

A neurological examination provided a detailed snapshot of the patient’s current clinical status. He was alert, oriented, and displayed normal memory, attention, and knowledge. Cranial nerve examinations did not yield any deficits. Motor examination demonstrated normal bulk and tone without evidence of pronator drift or fasciculations. Strength assessment indicated overall normal strength. Sensory evaluation revealed mildly reduced vibratory sense in the great toes bilaterally. Cerebellum and coordination assessments showed normal findings during finger-nose-finger testing bilaterally. Reflexes were within normal limits (2+).

## Discussion

Our patient had a history of chronic migraines, extremity weakness with exertion, and recurrent hemoptysis, coupled with a recent diagnosis of autism in his young daughter. Both the patient and his daughter experienced seizures in childhood. Such clinical pictures of both the patient and his daughter raised suspicion of a potential genetic etiology for the family's neurologic symptoms.

The initial workup with a brain MRI played a pivotal role in unraveling the diagnosis. In both the patient and his daughter, MRI revealed a confluent increased T2/FLAIR signal in the supratentorial deep white matter with basal ganglia involvement. Importantly, we observed an asymmetric hyperintensity pattern without indication of abnormal enhancement or evolving ischemia, which might otherwise fit with leukodystrophies. [1] Our patient displayed subcortical white matter involvement but lacked the parieto-occipital predominance that may be observed in Krabbe disease or X-linked adrenoleukodystrophy. [1] These imaging characteristics resemble MRI findings associated with known collagen vascular disorders. [2-4]

Hereditary collagen vascular disorders, particularly COL4A1 disorders, represent a group of genetic conditions with mutations predominantly affecting collagen proteins. These conditions are rare, with fewer than 100 families having been described as carrying the disease. [2] The COL4A1 gene encodes collagen type IV alpha 1, a crucial component of basement membranes in various tissues, including the cerebral vasculature. Mutations in COL4A1 are associated with cerebrovascular disease, leading to porencephaly, leukoencephalopathy, and, notably, cerebral small vessel disease, which can manifest as T2/FLAIR hyperintensities on brain MRI. [2,5] They are often autosomal dominant and fully penetrant mutations. There are three primary subtypes of COL4A1 disorders, which include autosomal dominant familial porencephaly, autosomal dominant brain small-vessel disease with hemorrhage, and hereditary angiopathy with nephropathy, aneurysms, and muscle cramps (HANAC) syndrome. [3,5] Mutations to the COL4A1/2 gene are known to cause childhood epilepsy and leukoencephalopathy, as were seen in our patient and his daughter. [2]

Analysis of copy number variation in adults with unexplained childhood-onset epilepsy and intellectual disability demonstrated COL4A1 as one of five genes with particular intolerance of variation and pathogenicity. [6] The mutation c.3698G>A (p.Gly1233Glu) in the COL4A1 gene is not only identified in the patient but also in his daughter, who has autism and pediatric epilepsy. Glycine residues are of particular importance in α-helix structures, where they serve in Gly-X-Y triple helix repeats and are thought to impart flexibility and facilitate branching in assembly. [7] The replacement of glycine, the simplest amino acid, with glutamine, which bears a bulkier side chain and proves less flexible for the formation of α-helices, is hypothesized to disrupt the secondary structure of the resulting collagen protein. [8,9] Upon review via the National Library of Medicine’s ClinVar tool, there are 202 published pathogenic variants of COL4A1; of these, 59 are missense mutations, and five of these involve substitutions of glycine with glutamine. Manifestations of these five missense mutations include brain small vessel disease without ocular abnormalities and autosomal dominant familial hematuria-retinal arteriolar tortuosity-contractures syndrome. [4,5]

The detection of mutation c.3698G>A (p.Gly1233Glu) in the COL4A1 gene of both the patient and his daughter raises compelling questions about its clinical significance as a collagen vascular disorder causing the visualized cerebrovascular changes and clinical manifestations. [5] Considering that this genetic variant is categorized as a VUS, further correlation with clinical findings is essential to elucidating its precise role in neurologic symptoms. The phenotypic variability of collagen vascular disorders underscores the complexity of such diagnoses and the necessity for a multidisciplinary approach encompassing clinical, genetic, and radiological assessments. The biological significance of the identified mutation, p.Gly1233Glu, in the context of cerebrovascular changes and its association with neurologic symptoms warrants further investigation to shed light on its clinical implications.

## Conclusions

This clinical presentation of a 44-year-old male patient, characterized by a history of pediatric epilepsy, chronic migraines, extremity weakness, and recurrent hemoptysis, in conjunction with neurologic symptoms in his daughter, led to brain imaging and genetic testing for a collagen vascular disorder.

Brain MRI findings in both the patient's and his daughter's parallel patterns were observed in established COL4A1 collagen vascular disorders, known for their diverse systemic manifestations. The identification of a VUS in the COL4A1 gene, specifically c.3698G>A (p.Gly1233Glu), in both the patient and his daughter raises compelling questions regarding its potential clinical significance. Given the association of COL4A1 gene mutations with cerebrovascular disease, porencephaly, leukoencephalopathy, and cerebral small vessel disease, the VUS may represent a novel pathogenic variant contributing to the family's neurologic symptoms. Specifically, it would most closely fit the autosomal dominant brain small-vessel disease variant of the COL4A1 disorders.

In summary, the VUS identified in the COL4A1 gene presents the possibility of being a novel pathogenic variant. This case underscores the complexity of diagnosing rare genetic conditions and the essential role of multidisciplinary approaches encompassing clinical, genetic, and radiological assessments. Further research and correlation with clinical findings are paramount to elucidating the precise clinical implications of this genetic variant.
